# Individual and combined effects of butyric acid glycerides and a multicomponent phytogenic supplement on growth performance, intestinal mucosal health, and immune response of broiler chickens under coccidiosis challenge

**DOI:** 10.1016/j.psj.2025.106046

**Published:** 2025-10-31

**Authors:** Hossein Ali Ghasemi, Hamidreza Nahandast, Iman Hajkhodadadi, Navid Nari, Kamran Taherpour

**Affiliations:** a Department of Animal Science, Faculty of Agriculture and Environment, Arak University, 38156-8-8349 Arak, Iran; b Department of Animal Science, Faculty of Agriculture, Ilam University, Ilam, Iran

**Keywords:** Butyrate glycerides, *Eimeria*-infected broilers, Intestinal health, Phytogenic additives, Productive performance

## Abstract

This study investigated the effects of butyric acid glycerides (BAG) and a multicomponent phytogenic product (MPH), both individually and in combination, on growth performance, carcass traits, hematological parameters, immune responses, gut morphology, and the expression of tight junction genes in broiler chickens challenged with coccidiosis. A total of 550 one-day-old male Ross 308 chicks were assigned to 5 treatment groups: negative control (NC; basal diet without challenge), positive control (PC; basal diet with coccidiosis challenge), PC supplemented with BAG (PC+BAG), PC supplemented with MPH (PC+MPH), and PC supplemented with both BAG and MPH (PC+BAG+MPH). The coccidial challenge, using a mixed *Eimeria* species inoculum, was administered on day 14. Results demonstrated that the combined PC+BAG+MPH treatment significantly improved final BW (2,798 g), ADG (65.6 g/day), and performance index (375) compared to the PC group (2,494 g, 58.3 g/day, and 289; *P* < 0.05), with values not significantly different from the NC group (2,926 g, 68.6 g/day, and 403; *P* > 0.05). Feed conversion ratio was significantly better in the PC+BAG+MPH (1.69) and NC (1.68) groups compared to the PC group (1.83; *P* < 0.05). Intestinal morphology was also significantly improved in the PC+BAG+MPH group, with increased villus height and villus height-to-crypt depth ratios in the duodenum, jejunum, and ileum (*P* < 0.05). All supplemented groups exhibited reduced oocyst excretion and lesion scores, with the greatest reductions observed in the PC+BAG+MPH group (*P* < 0.05). Additionally, this group showed increased serum IgG and IgA concentrations, upregulated expression of jejunal JAM-2, claudin, and ZO-1 genes, and a decreased heterophil-to-lymphocyte ratio compared to the PC group (*P* < 0.05). While the PC+BAG and PC+MPH treatments individually yielded improvements in certain growth and gut health parameters, these effects were less pronounced than those observed with the combined treatment. In conclusion, dietary supplementation with both BAG and MPH effectively mitigated the adverse effects of *Eimeria* challenge by improving growth performance, intestinal morphology, immune status, and mucosal barrier integrity in broiler chickens.

## Introduction

The broiler chicken industry, as a major source of animal protein, faces numerous challenges related to enteric diseases, particularly coccidiosis, which disrupts production performance and increases mortality (Amerah and Ravindran, 2015; Oxford and Selvaraj, 2019). Coccidiosis, a parasitic disease caused by various *Eimeria* species, damages the intestinal epithelium, reduces nutrient absorption, and induces inflammation, leading to decreased weight gain, poor feed conversion ratio, and increased mortality (Rostami et al., 2021; Bedford and Apajalahti, 2022). In addition to its direct effects on intestinal health, this disease accelerates the parasite transmission cycle and increases contamination within production units by elevating oocyst excretion and lesion scores (Nooreh et al., 2021; Javanmiri et al., 2024). Conventional control methods relying on anticoccidial drugs, although effective, have raised growing concerns regarding drug resistance and chemical residues, thereby increasing interest in natural and safe alternative solutions (Abbas et al., 2011; Broom, 2021).

Butyric acid, a short-chain fatty acid, plays a crucial role in maintaining intestinal mucosal health and enhancing performance in broiler chickens (Khan et al., 2022). It serves as the primary energy source for intestinal epithelial cells and supports digestive function by strengthening the mucosal barrier, reducing inflammation, and promoting tissue repair (Nari and Ghasemi, 2020; Mátis et al., 2022; Sizmaz et al., 2022). Protected forms of butyric acid, such as butyrate glycerides, have demonstrated enhanced efficacy in preserving mucosal integrity and upregulating the expression of tight junction proteins, owing to their targeted and gradual release within the intestine—particularly critical under infectious challenges such as coccidiosis (Ventura et al., 2021; Kpodo et al., 2025). Moreover, these compounds reduce mucosal injury, improve growth performance, and decrease mortality by modulating the intestinal microbiota and limiting the proliferation of parasites and opportunistic pathogens (Nari et al., 2020; Hansen et al., 2021). Recent studies indicate that butyrate glycerides help maintain gut health under environmental and infectious stress conditions and serve as promising alternatives to antibiotics in commercial poultry nutrition (Abbasi Arabshahi et al., 2021; Zhang et al., 2023).

Phytobiotics, defined as plant-derived bioactive compounds such as essential oils, flavonoids, tannins, and alkaloids, play a significant role in enhancing digestive health and production performance in poultry, particularly under coccidial challenge (Aljumaah et al., 2020; Ceylan et al., 2023). Owing to their antimicrobial and anti-inflammatory properties, these compounds reduce the burden of intestinal pathogens, including *Eimeria* parasites and opportunistic bacteria, while modulating the intestinal microbiota (Shahininejad et al., 2024; Galamatis et al., 2025a). Additionally, phytobiotics support the integrity of the intestinal mucosal barrier and facilitate recovery from inflammatory damage by stimulating both local and systemic immune responses and enhancing endogenous antioxidant levels (Jelveh et al., 2023; Galamatis et al., 2025b). Several studies have demonstrated that dietary supplementation with phytobiotics in coccidiosis-challenged poultry reduces the severity of intestinal lesions, improves performance metrics such as weight gain and feed conversion, and elevates immunoglobulin concentrations, thereby promoting overall flock health and productivity (Safavipour et al., 2022; Jiang et al., 2024). Consequently, phytobiotics, as natural and effective alternatives to antibiotics and coccidiostats, represent a promising strategy for managing intestinal diseases and enhancing production efficiency in the poultry industry.

Although natural supplements such as phytobiotics and organic acids have been investigated separately in several studies, there is limited and occasionally contradictory data on the synergistic effects of these two supplement groups and the precise mechanisms underlying their effects on intestinal health, immunity, and performance. It is hypothesized that the combination of butyrate glycerides with phytobiotics, due to the hydrophobic nature of phytochemicals, which increases bacterial membrane permeability and facilitates the entry of organic acids into pathogen cells, could enhance the effectiveness of these supplements and accelerate improvements in intestinal health and growth performance. Therefore, the present study aimed to investigate the single and combined effects of butyric acid glycerides and a mixed supplement of selected phytochemicals on growth performance, intestinal morphology, oocyst excretion rate, mucosal lesion severity, tight-junction protein gene expression, and immune and hematological parameters in broiler chickens challenged with coccidiosis.

## Materials and methods

### Birds and treatments

All animal care procedures and experimental protocols were approved by the Arak University Animal Care and Use Committee (approval number 1403-D-15807). To conduct this experiment, 550 one-day-old broiler chicks of the Ross 308 strain were randomly assigned to 5 treatment groups, each comprising 5 replicate pens with 22 chicks per replicate. An a priori power analysis for a one-way ANOVA with 5 treatments (α = 0.05) indicated at least 80 % power to detect an overall treatment effect at the pen level. On the first day, the chicks were weighed and randomly allocated to the treatment groups and replicates, ensuring that each replicate contained 22 chicks with a comparable average initial body weight of 42 ± 0.8 g. Throughout the 42-day rearing period, birds were housed in a fully controlled environment with ad libitum access to feed and water. The chicks were kept in floor pens measuring 2 × 1.75 m. Environmental conditions included maintaining the room temperature at 34 ± 1°C during the first week, followed by a gradual reduction to 23 ± 1°C. The lighting program consisted of 24 h of light and 0 h of darkness (24L:0D) for the first two days, followed by 23 h of light and 1 h of darkness (23L:1D) from day 3 onward.

The MPH used in this study was a commercial blend (Phytmax Cox; Phytosolutions S.L., Spain) comprising microencapsulated curcumin (turmeric extract; curcuminoids), capsicum oleoresin (capsaicin), cinnamaldehyde (from cinnamon), and piperine (Piper nigrum) in a lipid matrix to ensure stability and uniform dispersion. According to the certificate of analysis for the lot used (batch 15015300015), the finished product contained 0.28 % curcumin, 0.29 % capsaicin, 1.02 % cinnamaldehyde, and 0.045 % piperine. The butyric acid glycerides (BAG) used in this study (C4-BaBy) contained 25–35 % butyric acid monoglycerides, 50–55 % butyric acid diglycerides, and 15–25 % butyric acid triglycerides (SILO Company, Italy). This study employed a completely randomized design with 5 experimental treatments: (1) negative control group (NC; no coccidiosis infection + basal diet without supplementation), (2) positive control group (PC; coccidiosis-challenged + basal diet without supplementation), (3) the coccidiosis-challenged group with basal diet supplemented with 2 g/kg BAG (PC+BAG), (4) the coccidiosis-challenged group with basal diet supplemented with 200 mg/kg MPH (PC+MPH), and (5) the coccidiosis-challenged group with basal diet supplemented with both BAG and MPH (PC+BAG+MPH). The coccidiosis-challenged groups were inoculated on day 14 via oral gavage with a mixture of *Eimeria* parasites. The experimental diets were formulated according to the nutritional recommendations for each growth phase, as outlined in the Ross 308 Broiler Management Guide (Table 1).

**Table 1:** Ingredient composition and calculated nutrient contents of basal diets (as-fed basis).

Item	Starter (day 1 to 10)	Grower (day 11 to 24)	Finisher (day 25 to 42)
Ingredients (%)			
Corn	55.46	57.72	61.35
Soybean meal, 44 %	32.18	30.15	26.71
Corn gluten meal, 60 %	5.25	4.25	3.10
Soybean oil	2.20	3.50	4.80
Dicalcium phosphate	1.95	1.71	1.50
Limestone	1.16	1.07	1.00
Salt (NaCl)	0.22	0.23	0.2
Sodium bicarbonate	0.11	0.10	0.14
Vitamin premix^1^	0.25	0.25	0.25
Trace mineral premix^2^	0.32	0.32	0.32
DL-Methionine	0.26	0.23	0.22
L-Lysine HCl	0.43	0.32	0.28
L-Threonine	0.21	0.15	0.13
Calculated nutritive value			
Metabolizable energy, kcal/kg	3000	3100	3200
Crude protein (%)	23.0	21.5	19.5
Calcium (%)	0.96	0.87	0.79
Nonphytate phosphorus (%)	0.48	0.44	0.40
Sodium (%)	0.16	0.16	0.16
Digestible lysine (%)	1.28	1.15	1.03
Digestible methionine (%)	0.62	0.55	0.51
Digestible methionine + cysteine (%)	0.95	0.87	0.80
Digestible threonine (%)	0.86	0.77	0.69
DEB^3^, mEq/kg	250	240	230

1 Supplied per kg diet: 18 mg retinol, 4 mg cholecalciferol, 36 mg a-tocopherol acetate, 2 mg vitamin K_3_, 1.75 mg vitamin B_1_, 6.6 mg vitamin B_2_, 9.8 mg niacin, 29.65 mg pantothenic acid, 2.94 mg vitamin B_6_, 1 mg folic acid, 0.015 mg vitamin B_12_, 0.1 mg biotin, 250 mg choline chloride and 1 mg ethoxyquin.

2 Supplies per kg of the diet: Mn (manganese oxide), 120 mg; Zn (zinc sulfate), 100 mg; Fe (ferrous sulfate), 20 mg; Cu (cupric sulfate), 16 mg; I (potassium iodide), 1.2 mg; Se (sodium selenite), 0.32 mg.

3 DEB (dietary electrolyte balance) = (Na^+^, mEq/kg + *K*^+^, mEq/kg) – CL^−^, mEq/kg.

### Eimeria species challenge

All birds, except those in the NC group, received sporulated oocysts of *Eimeria* species via oral gavage. On day 14, each bird was administered 1 mL of a solution containing approximately 50,000, 10,000, and 5,000 oocysts of *Eimeria acervulina, Eimeria maxima*, and *Eimeria tenella*, respectively (Rostami et al., 2021). The oocysts of these three species were obtained from the Parasitology Laboratory at the University of Tehran (Tehran, Iran). They were collected from feces, litter, and intestinal samples of broiler chickens from commercial flocks in Iran. To facilitate sporulation, oocysts were maintained in potassium dichromate solution (Ghasemi et al., 2010). Broilers in the NC group, which were not challenged, received 1 mL of saline solution to account for management stress associated with gavage.

### Growth performance and slaughter traits

To calculate the average daily feed intake (ADFI) for each experimental unit, the amount of feed remaining at the end of each rearing stage was subtracted from the total feed provided during that period. On days 1, 10, 24, and 42, all chicks within each experimental unit were weighed to determine the average daily gain (ADG). The feed conversion ratio (FCR) was calculated by dividing the ADFI by the ADG of the chicks for each period. Throughout the experiment, the number and weight of mortalities were recorded and used to adjust the growth performance data for each experimental unit. Additionally, the European Performance Index (EPI) was calculated based on growth rate, survival rate, and FCR over the entire period (Ghasemi and Nari, 2023).

At 42 days of age, 2 birds were randomly selected from each replicate and slaughtered by severing the jugular vein. Following slaughter, the weights of the whole carcass, breast, and thigh were recorded. The yield of each part was calculated by dividing the weight of the respective part by the live weight prior to slaughter. After opening the abdominal cavity, the heart, liver, and lymphoid organs (spleen, bursa of Fabricius, and thymus) were excised and weighed. Additionally, abdominal fat deposits were collected and weighed. The relative weight of each organ or tissue was calculated by dividing the weight of each organ or tissue by the live weight of the bird.

### Coccidiosis lesion scoring and oocyst shedding

On day 21 (7 days after oocyst inoculation), 2 broilers from each pen were sacrificed to assess lesions associated with *Eimeria* challenge. The entire sections of the duodenum, ileum, and cecum were examined histologically for lesion assessment. Lesions were scored by a histopathologist blinded to the experimental design and graded on a scale from 0 (no visible lesions) to 4 (most severe lesions), following the established method of Johnson and Reid (1970).

Fresh excreta samples were collected in sample bags on days 6–8, 8–10, and 10–12 days post-infection (dpi) and carefully stored at 4°C for further processing. Oocyst shedding was measured according to a previous research study (Nooreh et al., 2021). Approximately 5 g of feces were accurately weighed and then added to a 50 mL centrifuge tube. The mixture was then vortexed thoroughly with 45 mL of saturated sodium chloride solution. After homogenization, stool samples were carefully loaded into a McMaster counting chamber. After 5 min at room temperature, oocysts were carefully examined under a microscope and the results were recorded as oocysts per gram of feces.

### Measurement of hematological parameters and blood leukocyte profile

On day 24 of the experiment, 2 chickens were randomly selected from each replicate (pen), and blood was collected from the wing vein. Blood samples were collected into tubes containing EDTA anticoagulant for hematological analysis. Red blood cell (RBC) and white blood cell (WBC) counts were determined using hemocytometry after dilution of the samples in Natt and Herrick solution. Hemoglobin concentration was measured using the cyanmethemoglobin method, and hematocrit percentage was determined by the microhematocrit method. To assess the differential leukocyte population, blood smears were prepared and stained with Giemsa stain. Using a light microscope (Olympus BX51, Tokyo, Japan), 100 leukocytes were counted per smear, and the percentages of heterophils, lymphocytes, monocytes, eosinophils, and basophils were recorded. The heterophil-to-lymphocyte ratio (H/L ratio) was calculated as an indicator of stress and immune response.

### Measurement of blood immunoglobulins

To assess blood immunoglobulin levels, serum samples were collected from 2 birds per replicate pen on day 13 (one day prior to *Eimeria* challenge) and day 24 (10 days post-challenge) by venipuncture of the wing veins. Blood samples were centrifuged at 2,500 × *g* for 15 min at 4°C to separate serum. Serum concentrations of immunoglobulins IgA, IgG, and IgM were measured using ELISA kits in 96-well flat-bottom plates. Quantitative ELISA kits specific for poultry IgA, IgG, and IgM (Bethyl Laboratories Inc., Montgomery, TX, USA) were used following the manufacturer’s protocols.

### Measurement of morphological parameters of small intestine

Measurement of morphological parameters of intestinal villi was conducted on day 24 of the experiment following sacrifice. Approximately 1-cm-long tissue samples were collected from the mid-sections of the duodenum, jejunum, and ileum (2 birds per replicate). Samples were fixed in formaldehyde, embedded in paraffin, and sectioned at 5 μm thickness. The sections were stained with Alcian blue, hematoxylin, and eosin, then examined under a light microscope (Olympus CX31, Shinjuku, Tokyo, Japan). Morphometric analysis was performed using QWinPlus software (version 3.1.0, Leica Cambridge Ltd., Cambridge, UK). Parameters assessed included villus height (VH), villus width (VW), crypt depth (CD), and villus absorptive surface area (VSA = 2π × (VW/2) × VH). Mean values from at least ten villi per sample were used for statistical analysis. Additionally, the number of goblet cells (GC) was determined by selecting 15 straight, and intact villi and their associated crypts and counting GC.

### Expression of tight junction protein genes

On day 24 of the experiment, jejunal samples were aseptically collected from broilers in each treatment group, immediately frozen in liquid nitrogen, and stored at −80°C until further analysis. Total RNA was extracted from the jejunum tissues using an RNA extraction kit (Pars Tous Com., Iran) according to the manufacturer’s instructions. The quality and quantity of the extracted RNA were assessed using a NanoDrop 2000 spectrophotometer (Thermo Fisher Scientific), with samples exhibiting an absorbance ratio (260/280 nm) between 1.8 and 2.0 considered suitable for downstream applications. Subsequently, 1 μg of RNA from each sample was reverse-transcribed into cDNA using a cDNA synthesis kit (Pars Tous, Iran). Quantitative real-time PCR (qPCR) was performed using gene-specific primers targeting claudin-1 (CLDN1), occludin (OCLN), zonula occludens-1 (ZO-1), and junctional adhesion molecule-2 (JAM-2), with primer sequences listed in Table 2. Amplifications were carried out on an ABI 7300 Real-Time PCR System (Applied Biosystems, Foster City, CA) using SYBR Green master mix (Pars Tous, Iran). Relative gene expression levels were calculated by the 2^−ΔΔCt^ method, using GAPDH as the internal reference gene.

**Table 2:** Primers for RT-qPCR analysis.

Gene^1^	Primer sequence (5°−3°)^2^	Length (nt)	GenBank number
OCLN	F: GGCCACCATGTTCAGCAAGAA	99	XM_046904540.1
	R: GACCCGTAGCCGTAATCAGCC		
CLDN1	F: AGAGGCATCAGGTATCTGGGT	214	NM_001013611.2
	R: CCCATCGAGAAGTAGGAGCCA		
JAM-2	F: GGATTCTGGGACCTACCGCTG	243	XM_046907882.1
	R: CTGCCTGTTCCTGTCTTTTCC		
ZO-1	F: CCCTGCCCGTGGGATGTTT	138	XM_040680630.2
	R: GCCCTGGCAGACATTTTGTTT		
GAPDH	F: CAGAACATCATCCCAGCGTCCAC	134	NM_204305.2
	R: CGGCAGGTCAGGTCAACAACAG		

1 OCLN = occludin; CLDN1 = claudin1; JAM-2 = junctional adhesion molecule 2; ZO-1 = zonula occluden p 1; GAPDH = glyceraldehyde-3-phosphate.

2 F = forward primer; *R* = reverse primer.

### Statistical analysis

The experimental unit for all outcomes was the pen. For carcass and physiological measurements, 2 birds per pen were randomly selected and their values averaged to generate a single pen-level observation. Data normality was assessed using the Shapiro-Wilk test prior to analysis. One-way analysis of variance (ANOVA) was performed to compare treatment means. When significant differences were identified, Tukey’s honestly significant difference (HSD) post hoc test was used for multiple comparisons. Intestinal lesion scores were analyzed at the pen level using the Kruskal–Wallis H test. Percentage data were arcsine-transformed to satisfy assumptions of normality and homogeneity of variance. In addition to the one-way analyses, a 2 × 2 factorial model was fitted on the challenged treatments (PC, PC+BAG, PC+MPH, PC+BAG+MPH) with fixed effects of BAG, MPH, and BAG × MPH, using pen-level data. Continuous outcomes were analyzed by two-way ANOVA. All statistical analyses were conducted using SAS software (version 9.4; SAS Institute Inc., Cary NC), with significance set at *P* < 0.05. Results are expressed as mean ± SEM.

## Results

### Growth performance

Table 3 summarizes the performance outcomes of broiler chickens challenged with an *Eimeria* species mixture up to 42 days of age. No significant differences were observed among treatments in BW, ADG, ADFI, or FCR up to 10 days of age (*P* > 0.05). However, BW at 24 and 42 days, as well as ADG from days 11 to 24 and over the entire 1-to-42-day period, were significantly greater in all supplemented groups compared to the PC group (*P* < 0.05). Moreover, ADG during the final phase (days 25 to 42) was significantly higher in the PC+BAG+MPH and NC groups relative to other treatments (*P* < 0.05). Among the coccidiosis-challenged groups, the PC+BAG+MPH treatment exhibited the highest BW at 42 days and overall ADG, with values statistically comparable to those of the NC group (*P* > 0.05) but significantly greater than those of the other challenged groups (*P* < 0.05).

**Table 3:** Effect of butyric acid glycerides (BAG) and a multicomponent phytogenic (MPH) on growth performance of broilers challenged with mixed *Eimeria* at 14 days of age.

	Experimental treatments^1^							*P*-value (2 × 2 factorial design)^2^		
Item	NC	PC	PC+BAG	PC+MPH	PC+BAG+MPH	SEM	*P*-value	BAG	MPH	BAG × MPH
Body weight (g)										
10 d	230.4	232.1	236.1	233.7	238.3	4.50	0.750	0.321	0.661	0.946
24 d	1123^a^	983^b^	1066^a^	1068^a^	1088^a^	14.3	<0.001	0.004	0.003	0.056
42 d	2926^a^	2494^c^	2625^b^	2660^b^	2798^a^	30.5	<0.001	0.001	<0.001	0.929
Average daily gain (g)										
d 1-10	18.61	18.80	19.19	18.95	19.40	0.462	0.777	0.349	0.661	0.945
d 11-24	63.77^a^	53.60^c^	59.27^b^	59.57^ab^	60.69^ab^	1.045	<0.001	0.007	0.004	0.054
d 25-42	100.17^a^	83.95^b^	86.62^b^	88.48^b^	95.00^a^	1.466	<0.001	0.008	0.001	0.227
d 1-42	68.62^a^	58.32^c^	61.45^b^	62.29^b^	65.56^a^	0.730	<0.001	0.001	<0.001	0.927
Average daily feed intake (g)										
d 1-10	22.11	22.16	23.32	22.34	22.22	0.354	0.131	0.184	0.230	0.104
d 11-24	92.02	85.61	87.47	88.92	88.66	1.506	0.082	0.610	0.161	0.498
d 25-42	184.5^a^	169.9^c^	174.7^bc^	179.8^ab^	176.6^bc^	1.641	<0.001	0.631	0.003	0.029
d 1-42	115.0^a^	106.6^c^	109.6^bc^	112.0^b^	110.5^b^	0.698	<0.001	0.317	<0.001	0.007
Feed conversion ratio										
d 1-10	1.19	1.18	1.22	1.18	1.15	0.031	0.642	0.896	0.274	0.274
d 11-24	1.44^b^	1.60^a^	1.48^ab^	1.50^ab^	1.46^ab^	0.034	0.032	0.045	0.104	0.242
d 25-42	1.84^b^	2.02^a^	2.02^a^	2.04^a^	1.86^b^	0.034	0.001	0.026	0.065	0.037
d 1-42	1.68^b^	1.83^a^	1.78^a^	1.80^a^	1.69^b^	0.019	<0.001	0.001	0.007	0.118
Mortality (d 1-42, %)	2.96^c^	11.11^a^	7.41^ab^	6.67^bc^	5.19^bc^	0.966	<0.001	0.021	0.005	0.291
EPI (d 1-42)	403.4^a^	288.9^c^	311.0^bc^	329.0^b^	374.8^a^	8.205	<0.001	0.001	<0.001	0.188

Means within a row not sharing the same superscript are different at *P* < 0.05. Values are means of 5 replicates (pens) per treatment and 22 chickens per replicate.

1 NC (negative control), unchallenged group fed basal diet without any additives. PC (positive control) Eimeria-challenged group fed basal diet without any additives; PC+BAG, PC supplemented with butyric acid glycerides; PC+MPH, PC supplemented with a multicomponent phytogenic supplement; PC+BAG+MPH, PC supplemented with butyric acid glycerides and a multicomponent phytogenic supplement.

2 To evaluate interaction between additives, a 2 × 2 factorial model was fitted to the challenged treatments (PC, PC+BAG, PC+MPH, PC+BAG+MPH).

All experimental treatments, except for PC+MPH, showed reduced ADFI compared to the NC group during days 25 to 42 (*P* < 0.05). Across the entire experimental period, all challenged groups consumed less feed than the NC group (*P* < 0.05). However, within the challenged treatments, the PC+MPH and PC+BAG+MPH groups had significantly higher ADFI than the PC group (*P* < 0.05). The FCR from days 11 to 24 was significantly lower in the NC group compared to the PC group (*P* < 0.05). During days 25 to 42 and over the entire experimental period, both the NC and PC+BAG+MPH groups maintained significantly improved FCR relative to other treatments (*P* < 0.05).

Mortality rates in the PC+MPH and PC+BAG+MPH treatments were comparable to those in the NC group (*P* > 0.05) and significantly lower than those observed in the PC group (*P* < 0.05). Mortality in the PC+BAG treatment was intermediate, differing significantly only from the NC group (*P* < 0.05). The performance index was higher in the NC, PC+MPH, and PC+BAG+MPH groups compared to the PC group, with the greatest values observed in the NC and PC+BAG+MPH treatments (*P* < 0.05). Based on the factorial design among challenged groups, there were significant BAG × MPH interaction effects in terms of BW at 24 days (*P* = 0.056), ADG from days 11 to 24 (*P* = 0.054), ADFI during days 25 to 42 (*P* = 0.029) and over the entire experimental period (*P* = 0.007), and FCR from days 25 to 42 (*P* = 0.037).

### Slaughter variables

Slaughter traits at 42 days of age are presented in Table 4. The results demonstrated that breast meat yield was significantly higher, while relative liver weight was significantly lower, in the NC, PC+MPH, and PC+BAG+MPH treatments compared to the PC treatment (*P* < 0.05). Additionally, breast meat yield was reduced in the PC treatment relative to the NC group (*P* < 0.05). The percentage of abdominal fat was significantly lower in all supplemented groups compared to the PC treatment (*P* < 0.05). In contrast, no significant differences were observed among treatments for thigh meat yield or the relative weights of the heart, spleen, thymus, and bursa of Fabricius (*P* > 0.05). In factorial analysis, significant interaction effects were observed between BAG and MPH on abdominal fat percentage (*P* = 0.008).

**Table 4:** Effect of butyric acid glycerides (BAG) and a multicomponent phytogenic (MPH) on carcass traits of 42-days-old broiler chickens following mixed *Eimeria* species challenge at 14 days of age.

	Experimental treatments^1^							*P*-value (2 × 2 factorial design)^2^		
Item^3^	NC	PC	PC +BAG	PC+MPH	PC+BAG+MPH	SEM	*P*-value	BAG	MPH	BAG × MPH
Carcass	74.21^a^	71.49^b^	72.74^ab^	73.59^a^	73.53^a^	0.421	0.002	0.197	0.005	0.163
Breast	28.69^a^	26.19^b^	27.83^ab^	27.31^ab^	27.81^ab^	0.420	0.007	0.031	0.239	0.215
Thigh	25.11	23.74	24.48	24.87	24.51	0.625	0.608	0.779	0.391	0.420
Abdominal fat	0.94^b^	1.49^a^	1.10^b^	1.09^b^	1.06^b^	0.054	<0.001	0.003	0.002	0.008
Liver	1.78^c^	2.22^a^	2.05^ab^	1.88^bc^	1.94^bc^	0.064	0.001	0.481	0.004	0.107
Heart	0.500	0.586	0.523	0.471	0.500	0.035	0.239	0.666	0.096	0.258
Spleen	0.112	0.151	0.120	0.126	0.123	0.010	0.088	0.086	0.244	0.162
Thymus	0.285	0.272	0.305	0.308	0.289	0.019	0.629	0.732	0.610	0.202
Bursa	0.149	0.124	0.123	0.147	0.132	0.010	0.202	0.447	0.125	0.471

Means within a row not sharing the same superscript are different at *P* < 0.05. Values are means of 5 replicates (pens) per treatment and 2 chickens per replicate.

1 NC (negative control), unchallenged group fed basal diet without any additives. PC (positive control) Eimeria-challenged group fed basal diet without any additives; PC+BAG, PC supplemented with butyric acid glycerides; PC+MPH, PC supplemented with a multicomponent phytogenic supplement; PC+BAG+MPH, PC supplemented with butyric acid glycerides and a multicomponent phytogenic supplement.

2 To evaluate interaction between additives, a 2 × 2 factorial model was fitted to the challenged treatments (PC, PC+BAG, PC+MPH, PC+BAG+MPH).

3 Based on preslaughter live body weight.

### Hematology and leukocyte profile

The results pertaining to hematological parameters and the leukocyte profile in 24-day-old broiler chickens are summarized in Table 5. RBC count and hemoglobin concentration were significantly decreased in the PC and PC+BAG treatments compared to the NC group (*P* < 0.05). Hematocrit percentage was elevated in all experimental treatments relative to the PC group, with the highest values observed in the NC group (*P* < 0.05). The PC+BAG+MPH treatment, similar to the NC group, exhibited a lower heterophil percentage, higher lymphocyte percentage, and consequently a reduced H/L ratio compared to the PC treatment (*P* < 0.05). The percentage of blood eosinophils was increased in the PC and PC+BAG groups relative to the NC group (*P* < 0.05), whereas treatments supplemented with the phytogenic product (PC+MPH and PC+BAG+MPH) showed intermediate values that did not differ significantly from other groups (*P* > 0.05). Total WBC count, as well as the percentages of monocytes and basophils, were unaffected by the experimental treatments (*P* > 0.05). Based on the factorial design analysis, the interactive effect between BAG and MPH was observed only on hematocrit percentage (*P* = 0.037).

**Table 5:** Effect of Effect of butyric acid glycerides (BAG) and a multicomponent phytogenic (MPH) on hematology and leukocyte profile of 24-days-old broiler chickens following mixed *Eimeria* species challenge at 14 days of age.

	Experimental treatments^1^							*P*-value (2 × 2 factorial design)^2^		
Item^3^	NC	PC	PC+BAG	PC+MPH	PC+BAG+MPH	SEM	*P*-value	BAG	MPH	BAG × MPH
RBC (× 10^6^/µL)	2.68^a^	1.82^b^	2.07^b^	2.21^ab^	2.19^ab^	0.120	0.001	0.331	0.042	0.244
WBC (× 10^3^/µL)	20.97	22.05	22.90	21.96	21.99	1.125	0.820	0.700	0.662	0.700
Hb (mg/dL)	15.73^a^	11.20^b^	11.88^b^	12.83^ab^	12.85^ab^	0.795	0.007	0.630	0.083	0.650
PCV (%)	32.4^a^	20.0^c^	26.4^b^	26.2^b^	27.8^b^	1.05	<0.001	0.002	0.002	0.037
Leukocyte profile										
Heterophils (H)	24.00^b^	32.00^a^	27.60^ab^	26.00^ab^	25.40^b^	1.491	0.012	0.123	0.017	0.234
Lymphocytes (L)	72.20^a^	61.60^b^	66.40^ab^	66.40^ab^	69.80^a^	1.534	0.001	0.021	0.021	0.667
Eosinophile	1.60^b^	4.00^a^	3.80^a^	3.60^ab^	2.40^ab^	0.514	0.014	0.172	0.085	0.323
Monocyte	1.20	1.20	1.20	2.00	0.80	0.438	0.431	0.204	0.665	0.204
Basophile	1.00	1.20	1.00	2.00	1.80	0.420	0.322	0.657	0.089	1.000
H:L ratio	0.334^b^	0.527^a^	0.418^ab^	0.393^ab^	0.365^b^	0.0320	0.004	0.060	0.013	0.248

Means within a row not sharing the same superscript are different at *P* < 0.05. Values are means of 5 replicates (pens) per treatment and 2 chickens per replicate.

1 NC (negative control), unchallenged group fed basal diet without any additives. PC (positive control) Eimeria-challenged group fed basal diet without any additives; PC+BAG, PC supplemented with butyric acid glycerides; PC+MPH, PC supplemented with a multicomponent phytogenic supplement; PC+BAG+MPH, PC supplemented with butyric acid glycerides and a multicomponent phytogenic supplement.

2 To evaluate interaction between additives, a 2 × 2 factorial model was fitted to the challenged treatments (PC, PC+BAG, PC+MPH, PC+BAG+MPH).

3 RBC, red blood cells; WBC, white blood cells; Hb, hemoglobin; PCV, packed cell volume (Hematocrit).

### *Intestinal lesion score* and excreta oocyst shedding

**Figure 1:**
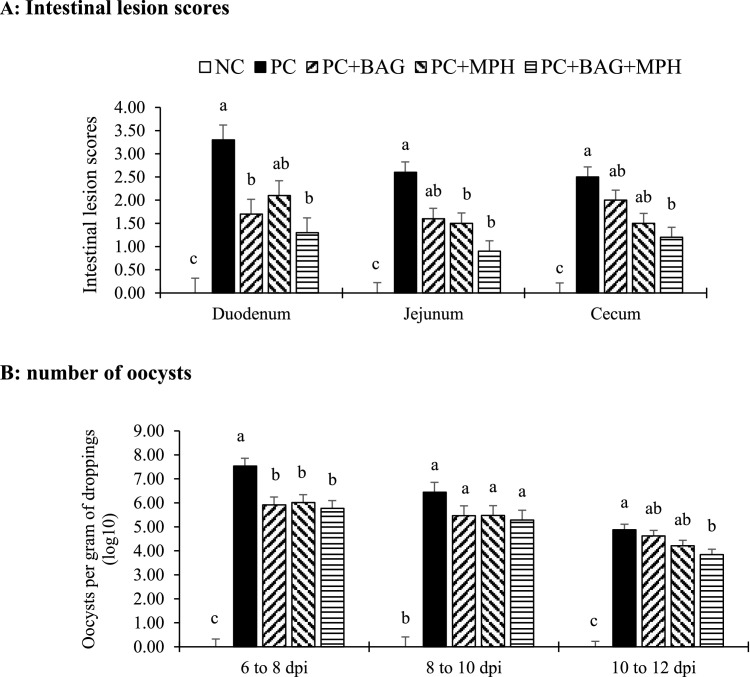
Intestinal lesion scores (A; 6 days after initial infection) and number of oocysts per gram of droppings (B; 6 to 12 days after initial infection) observed in broiler chickens after mixed *Eimeria* species challenge at 14 days of age. Different letters within the same histogram indicate significant differences among groups according to Tukey's multiple-range test (*P* < 0.05). Values are means of 5 replicates (pen) per treatment. The Kruskal-Wallis nonparametric statistical test was used for intestinal lesion scores. The factorial design analysis among challenged groups showed no interaction between BAG and MPH in relation to intestinal lesion scores (*P* > 0.05).

Abbreviation: NC (negative control), unchallenged group fed basal diet without any additives. PC (positive control) *Eimeria*-challenged group fed basal diet without any additives; PC+BAG, PC supplemented with butyric acid glycerides; PC+MPH, PC supplemented with a multicomponent phytogenic supplement; PC+BAG+MPH, PC supplemented with butyric acid glycerides and a multicomponent phytogenic supplement.

The effects of treatments on intestinal lesion scores and oocyst excretion rates in feces are illustrated in Figure 1. Regarding intestinal lesion scores (Figure 1A), no lesions were observed in any intestinal segment of the NC group (*P* > 0.05). All supplemented treatments, except PC+MPH, significantly reduced the severity of duodenal lesions caused by *Eimeria acervulina* compared to the PC group (*P* < 0.05). In the jejunum, treatments containing phytogenic supplements (PC+MPH and PC+BAG+MPH) resulted in significantly lower lesion scores associated with *Eimeria maxima* infection compared to the PC group (*P* < 0.05). In the cecum, the PC+BAG+MPH group exhibited a significantly reduced lesion score caused by *Eimeria tenella* infection relative to the PC group (*P* < 0.05).

No oocyst shedding was detected in the NC group across all sampling days (Figure 1B). Among the coccidiosis-challenged groups, all supplemented treatments significantly reduced oocyst excretion compared to the PC group between days 6 and 8 post-infection. Between days 8 and 10 post-infection, oocyst excretion rates were statistically similar among all supplemented groups (*P* > 0.05). However, between days 10 and 12 post-infection, the combined supplementation treatment (PC+BAG+MPH) exhibited a significantly lower oocyst excretion rate than the PC group (*P* < 0.05). In the factorial design analysis, the interactive effects of BAG and MPH on the intestinal lesion scores, as well as oocyst excretion rates, were not significant (*P* > 0.05)

### Serum immunoglobulins

The results related to blood immunoglobulin concentrations on day 13 (prior to coccidiosis challenge) and day 24 (10 days post-challenge) are presented in Table 6. On day 13, concentrations of IgG and IgA were not significantly affected by the experimental treatments. In contrast, IgM levels were significantly higher in the PC+MPH treatment compared to both the PC and NC groups (*P* < 0.05). On day 24, IgG and IgM concentrations were elevated in all *Eimeria*-challenged treatments relative to the NC group (*P* < 0.05). Among the challenged groups, the highest IgG concentration was observed in the PC+BAG+MPH treatment, which differed significantly from the PC and PC+BAG treatments (*P* < 0.05). Additionally, treatments containing the phytogenic supplement (PC+MPH and PC+BAG+MPH) exhibited the highest IgA concentrations, significantly surpassing those in the PC group (*P* < 0.05). A trend toward increased IgM concentration (*P* = 0.06) was also noted in the PC+MPH and PC+BAG+MPH treatments compared to other experimental groups. In the factorial design analysis, there was a BAG × MPH interaction effect in terms of serum IgA concentration at day 13 (*P* = 0.006).

**Table 6:** Effect of butyric acid glycerides (BAG) and a multicomponent phytogenic (MPH) on serum levels of immunoglobulins (ng/mL) observed at 13 and 24 days of age in broiler chickens infected with a mixture of *Eimeria* species at 14 days of age.

	Experimental treatments^1^							*P*-value (2 × 2 factorial design)^2^		
Item	NC	PC	PC +BAG	PC+MPH	PC+BAG+MPH	SEM	*P*-value	BAG	MPH	BAG × MPH
Day 13										
IgG	240.0	232.1	235.4	244.9	241.1	10.33	0.915	0.983	0.365	0.724
IgM	64.7^ab^	61.7^b^	76.7^ab^	80.1^a^	73.7^ab^	3.77	0.010	0.066	<0.001	0.488
IgA	89.4	97.8	107.0	113.8	108.7	11.31	0.578	0.225	0.037	0.006
Day 24										
IgG	289.1^d^	989.6^c^	1081.4^bc^	1204.5^ab^	1247.5^a^	33.21	<0.001	0.907	0.007	0.646
IgM	106.7	101.5	105.5	125.4	123.1	6.69	0.060	0.871	0.473	0.562
IgA	110.7^c^	368.6^b^	409.2^ab^	431.8^a^	428.6^a^	11.92	<0.001	0.155	0.005	0.099

Means within a row not sharing the same superscript are different at *P* < 0.05. Values are means of 5 replicates (pens) per treatment and 2 chickens per replicate.

1 NC (negative control), unchallenged group fed basal diet without any additives. PC (positive control) Eimeria-challenged group fed basal diet without any additives; PC+BAG, PC supplemented with butyric acid glycerides; PC+MPH, PC supplemented with a multicomponent phytogenic supplement; PC+BAG+MPH, PC supplemented with butyric acid glycerides and a multicomponent phytogenic supplement.

2 To evaluate interaction between additives, a 2 × 2 factorial model was fitted to the challenged treatments (PC, PC+BAG, PC+MPH, PC+BAG+MPH).

### Gut morphological parameters

**Figure 2:**
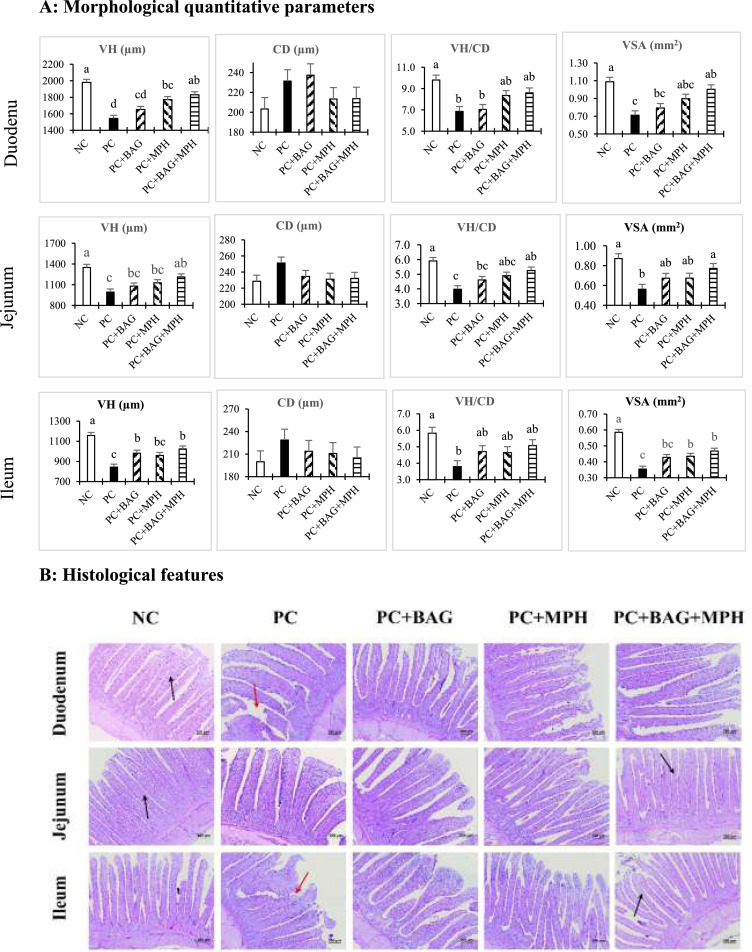
The morphological quantitative parameters and histological features (based on Alcian blue, hematoxylin, and eosin staining) of the mucosa of the duodenum, jejunum, and ileum in 24-days-old broiler chickens following a mixed *Eimeria* species challenge at 14 days of age. Different letters within the same histogram indicate significant differences among groups according to Tukey's multiple-range test (*P* < 0.05). The factorial design analysis among challenged groups showed no interaction between BAG and MPH in relation to intestinal morphological quantitative parameters (*P* > 0.05). For histological observation, images at a lower magnification (100 ×) are provided. Black arrows indicate complete and developed villus structures, while red arrows denote incomplete and shorter ones.

Abbreviation: NC (negative control), unchallenged group fed basal diet without any additives. PC (positive control) *Eimeria*-challenged group fed basal diet without any additives; PC+BAG, PC supplemented with butyric acid glycerides; PC+MPH, PC supplemented with a multicomponent phytogenic supplement; PC+BAG+MPH, PC supplemented with butyric acid glycerides and a multicomponent phytogenic supplement.

The results pertaining to the morphological traits of different sections of the small intestine in broiler chickens at 24 days of age are presented in Figure 2. In the duodenum, VH was significantly increased in the NC, PC+MPH, and PC+BAG+MPH treatments compared to the PC treatment (*P* < 0.05). The VH/CD ratio was elevated in the NC treatment compared to both the PC and PC+BAG treatments (*P* < 0.05). Additionally, VSA was significantly greater in the NC and PC+BAG+MPH groups relative to the PC group (*P* < 0.05). In the jejunum, the NC and PC+BAG+MPH treatments significantly increased VH, VH/CD ratio, and VSA compared to the PC treatment (*P* < 0.05). In the ileum, all experimental treatments, except for PC+MPH, significantly increased VH relative to the PC group, with the highest VH observed in the NC treatment (*P* < 0.05). Furthermore, the VH/CD ratio in the ileum was significantly greater in the NC treatment compared to the PC group (*P* < 0.05). The VSA in the ileum was significantly increased in all treatments except for the PC+BAG group when compared to the PC treatment (*P* < 0.05). In the factorial analysis, no significant interaction effects were observed between BAG and MPH on any of the intestinal morphological traits (*P* > 0.05).

**Figure 3:**
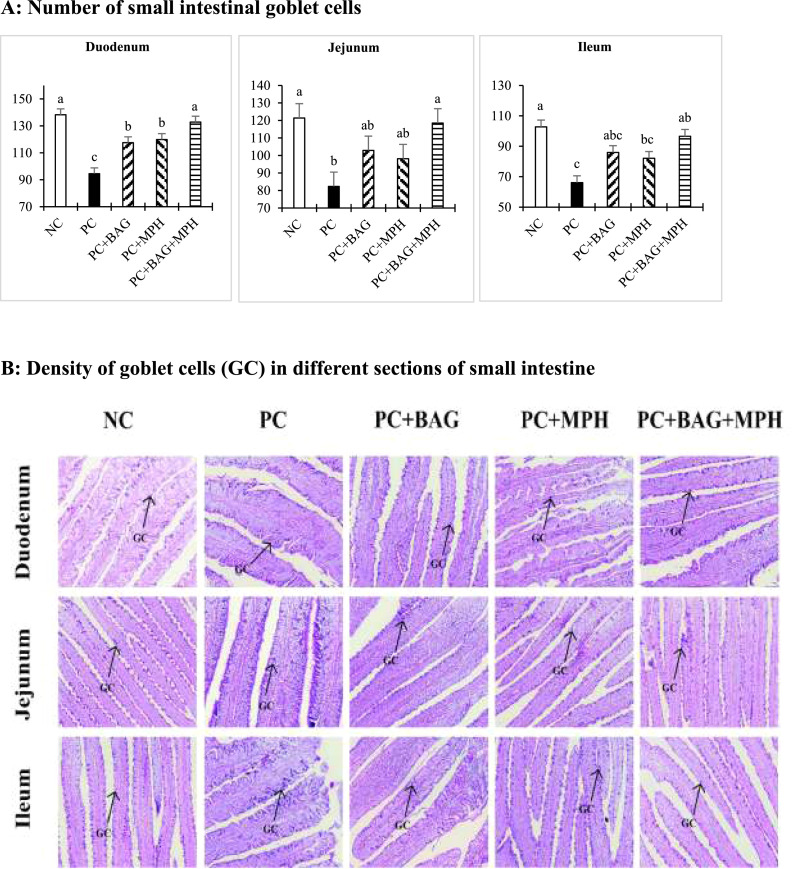
The number of small intestinal goblet cells (GC) and histological images in 24-days-old broiler chickens following a mixed *Eimeria* species challenge at 14 days of age. Different letters within the same histogram indicate significant differences among groups according to Tukey's multiple-range test (*P* < 0.05). The factorial design analysis among challenged groups showed no interaction between BAG and MPH in relation to intestinal goblet cells (*P* > 0.05). For histological observation, images at a lower magnification (100 ×) are provided.

Abbreviation: NC (negative control), unchallenged group fed basal diet without any additives. PC (positive control) Eimeria-challenged group fed basal diet without any additives; PC+BAG, PC supplemented with butyric acid glycerides; PC+MPH, PC supplemented with a multicomponent phytogenic supplement; PC+BAG+MPH, PC supplemented with butyric acid glycerides and a multicomponent phytogenic supplement.

According to the GC counts in different sections of the small intestine (Figure 3), all experimental diets resulted in higher duodenal GC numbers relative to the PC diet (*P* < 0.05), with the NC and PC+BAG+MPH treatments exhibiting the highest GC counts (*P* < 0.05). In the jejunum and ileum, GC numbers were also greater in the NC and PC+BAG+MPH treatments compared to the PC group (*P* < 0.05). No significant interaction effects between BAG and MPH on intestinal GC counts were observed (*P* > 0.05).

### Gene expression of tight junction proteins

**Figure 4:**
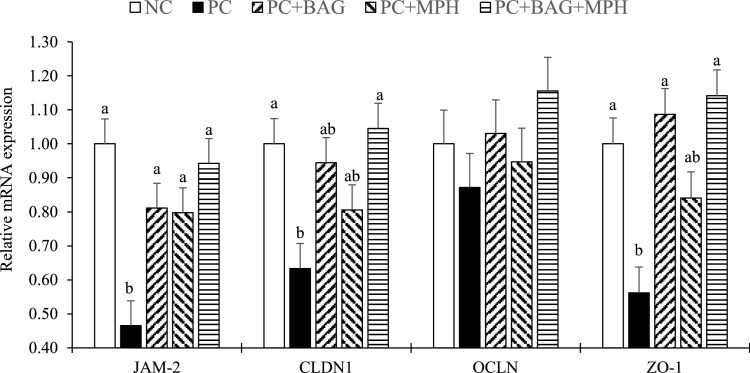
Bar charts of jejunal mRNA expression levels of junctional adhesion molecule 2 (JAM-2), claudin 1 (CLDN1), occludin (OCLN), and zonula occludens 1 (ZO-1) in 24-days-old broiler chickens following a mixed *Eimeria* species challenge at 14 days of age. Different letters within the same histogram indicate significant differences among groups according to Tukey's multiple-range test (*P* < 0.05). Values are means of 5 replicates (pen) per treatment and 2 chickens per replicate. The factorial design analysis among challenged groups showed no interaction between BAG and MPH in relation to jejunal mRNA expression levels of tight-junction proteins (*P* > 0.05).

Abbreviation: NC (negative control), unchallenged group fed basal diet without any additives. PC (positive control) Eimeria-challenged group fed basal diet without any additives; PC+BAG, PC supplemented with butyric acid glycerides; PC+MPH, PC supplemented with a multicomponent phytogenic supplement; PC+BAG+MPH, PC supplemented with butyric acid glycerides and a multicomponent phytogenic supplement.

The results of jejunal tight junction protein gene expression in broilers at 24 days of age, challenged with a mixture of *Eimeria* parasites, are presented in Figure 4. Expression of the JAM-2 gene was significantly higher in all experimental treatments compared to the PC group (*P* < 0.05). The PC+BAG+MPH treatment, similar to the NC group, significantly upregulated jejunal CLDN1 expression relative to the PC group (*P* < 0.05). Additionally, a significant increase in ZO-1 expression was observed in broilers fed diets containing BAG (PC+BAG and PC+BAG+MPH) compared to the PC group (*P* < 0.05). No significant differences were detected among treatments in the expression of OCLN (*P* > 0.05). In the factorial analysis, no significant interaction effects were observed between BAG and MPH on gene expression of jejunal tight junction proteins (*P* > 0.05).

## Discussion

The findings of this study demonstrate that broiler chickens challenged with a mixture of *Eimeria* species exhibited significantly improved growth performance when supplemented with butyric acid glycerides and a multicomponent phytogenic supplement, particularly in the combined treatment (PC+BAG+MPH). This treatment resulted in the highest final BW, ADG, and performance index, alongside the lowest FCR and mortality among the challenged groups, suggesting that the combined group showed the largest improvement in growth performance under a coccidiosis challenge. The observed improvements in intestinal health and immune responses in the respective groups likely contributed collectively to these growth benefits. These findings are consistent with previous studies showing that phytogenic supplements, rich in bioactive compounds with antimicrobial, antioxidant, and anti-inflammatory properties, can mitigate the adverse effects of coccidiosis (Galamatis et al., 2025a; Park et al., 2023). Phytogenic compounds such as cinnamaldehyde and curcumin have been shown to strengthen the intestinal mucosal barrier and enhance nutrient absorption (Ruan et al., 2019; Yang et al., 2021), while capsaicin supports digestive health through its anti-inflammatory and antioxidant effects (Liu et al., 2021). Piperine may further potentiate the efficacy of these compounds by increasing their bioavailability (Shi et al., 2020). Beyond direct epithelial effects, phytogenics can beneficially modulate the cecal microbiome and, in turn, support resistance to coccidial damage and nutrient capture. Previous studies have also shown that phytochemical supplements suppress opportunistic pathogens (e.g., *Clostridium perfringens*), enrich lactobacilli and other SCFA-producing taxa, and upregulate digestive and barrier genes, mechanisms linked to improved nutrient absorption and growth performance (Lillehoj et al., 2018; Abdelli et al., 2021; Chen et al., 2024). Additionally, protected butyric acid serves as a crucial energy source for intestinal epithelial cells, promoting mucosal integrity and inhibiting pathogenic bacteria, thereby improving FCR and reducing mortality in challenged chickens (Sadurní et al., 2022; Sizmaz et al., 2022; Khatami et al., 2024).

The study also observed a physiological response to infection, manifested as reduced feed intake in the challenged groups compared to the negative control. However, the relative increase in ADFI in the phytogenic-supplemented groups (PC+MPH and PC+BAG+MPH) suggests improved appetite and greater tolerance to challenge conditions. Moreover, the reduction in mortality rates among the supplemented groups, relative to the PC group, highlights the protective effects of these compounds on poultry health under coccidiosis stress, corroborating findings from previous studies (Tsiouris et al., 2021; Nouri, 2023). The performance index, which integrates BW, FCR, and survival rate, serves as a comprehensive measure of productivity in the poultry industry. The improvements observed in the phytogenic- and butyric acid–supplemented groups can be attributed to reduced mortality and enhanced feed efficiency, reflecting lower metabolic stress and improved immune and nutritional status in the birds. It is also important to note that, while controlled conditions allowed precise estimation of treatment effects, the magnitude of benefit from BAG and MPH may vary under commercial variability (e.g., temperature–humidity index, litter moisture, stocking density, coccidiosis control program, diet matrix, and baseline health).

In this study, it was observed that the NC, the phytogenic supplement, and the combination of butyrate glycerides and phytogenic supplement significantly increased breast meat yield and reduced relative liver weight compared to the PC treatment. These results highlight the beneficial effects of these supplements in maintaining bird health and improving carcass quality under the stressful conditions of coccidial infection. The reduced breast meat yield observed in the PC group compared to the NC group is attributable to intestinal mucosal damage and impaired nutrient absorption caused by coccidiosis, as reported in previous studies (Alqhtani et al., 2023a, 2023b). The decreased relative liver weight in the supplemented groups may also indicate a reduction in inflammatory responses or infection-induced damage, which is associated with improved metabolic status and regulation of immune function (Alqhtani et al., 2023a; Nouri, 2023). Conversely, the reduction in abdominal fat percentage observed across all supplemented treatments compared to the PC group suggests enhanced regulation of lipid metabolism and reduced oxidative stress, effects likely mediated by active compounds in the phytogenic supplement such as cinnamaldehyde (Chen et al., 2024) and curcumin (Yang et al., 2023), as well as by the anti-inflammatory and gut health–promoting properties of protected butyric acid (Khatami et al., 2024). Importantly, the combined effects observed in the PC+BAG+MPH treatment led to further improvements in carcass traits, underscoring the significance of combining plant-derived compounds and protected organic acids in managing pathogenic challenges such as coccidiosis in poultry production. These findings align with those of previous studies, which reported improvements in metabolic and health status parameters following supplementation with phytochemical compounds (Prihambodo et al., 2021; Arisha et al., 2024) and protected butyric acid (Proszkowiec-Weglarz et al., 2020; Kpodo et al., 2025).

Regarding hematology and the blood leukocyte profile, the results demonstrated that RBC count and hemoglobin concentration were significantly reduced in the PC group compared to the NC group, indicating a detrimental effect of coccidiosis infection on hematological status and the oxygen-carrying capacity of the blood (Arczewska-Włosek et al., 2018; Cowieson et al., 2020). This reduction may be attributed to intestinal mucosal damage, impaired nutrient absorption, and inflammatory stress induced by infection, all of which adversely affect erythropoiesis (Lei et al., 2022). In contrast, supplementation with a combination of phytogenic compounds and protected butyric acid produced significant beneficial effects by modulating hematological parameters and systemic immune responses in broilers under a coccidiosis challenge. This combination appears to support health maintenance and potentially enhance disease resistance by reducing systemic stress, as evidenced by a decreased H/L ratio, and by improving oxygen transport metrics (RBC count and hemoglobin concentration). These findings are consistent with previous reports highlighting the positive role of phytogenic and organic acid supplements in preserving hematological health and mitigating oxidative stress in poultry (Makled et al., 2019; Akue et al., 2024; Chahar et al., 2024). A reduction in the H/L ratio reflects diminished systemic stress and a more balanced inflammatory response in birds (Maxwell, 1993). Prior studies have documented the antioxidant, anti-inflammatory, and antimicrobial properties of phytogenic compounds such as cinnamaldehyde and curcumin (Yang et al., 2023; Chen et al., 2024), along with the role of butyric acid in maintaining intestinal mucosal integrity and enhancing immune function (Nari et al., 2020; Melaku et al., 2021).

The results of this study demonstrated that, in coccidiosis-challenged groups, all supplemented treatments significantly reduced oocyst excretion in the feces and lesion severity in various intestinal segments compared to the PC group. These findings clearly indicate that phytogenic supplements and butyric acid, particularly in the combined treatment (PC+BAG+MPH), effectively mitigate the severity of infection and parasite oocyst shedding. This aligns with previous research showing reduced oocyst excretion resulting from a lowered parasite burden and enhanced immune competence conferred by phytogenic compounds (Arczewska-Włosek et al., 2018; Chang et al., 2021; Das et al., 2021) or by butyric acid (Proszkowiec-Weglarz et al., 2020; Wang et al., 2021). The reduction in oocyst shedding in the supplemented groups likely results from their modulatory effects on the intestinal environment, including diminished inflammation and tissue damage caused by infection. Butyric acid, owing to its anti-inflammatory properties, attenuates intestinal mucosal inflammation and damage induced by coccidiosis, thereby creating an unfavorable environment for parasite proliferation (Sizmaz et al., 2022). Furthermore, phytogenic supplements, through their antioxidant and antimicrobial activities, enhance host responses to infection by reducing pathogen load and strengthening immune defenses (Park et al., 2023).

The activation of immune function in response to coccidiosis is a critical adaptive mechanism (Mustafa et al., 2021). Upon *Eimeria* infection, the host’s immune system is stimulated to produce various immunoglobulins, including IgG, IgA, and IgM, which play pivotal roles in identifying and neutralizing the pathogen (Perez-Carbajal et al., 2010). The observed increase in these immunoglobulins post-challenge indicates an active immune response aimed at combating the infection (Nooreh et al., 2021; Rostami et al., 2021). This adaptive response is essential for controlling parasite proliferation and mitigating intestinal damage (Girard et al., 1997). The enhanced immunoglobulin levels in broilers receiving phytogenic supplements and butyric acid suggest that these additives may potentiate the host’s natural defense mechanisms, leading to a more robust and effective immune response against coccidiosis. Notably, among the challenged treatments, the combined phytogenic and butyric acid supplementation (PC+BAG+MPH) induced the highest IgG and IgA concentrations, which were significantly greater than those observed in the PC group. This pronounced immune response likely reflects the combined effects of phytogenics and butyric acid in augmenting immune function and enhancing the anti-*Eimeria* immune response, as reported in previous studies involving butyric acid and phytogenic supplements (Makled et al., 2019; Abd El-Ghany, 2020; Seidavi et al., 2023). Mechanistically, butyrate can signal via GPR41/43 and through histone deacetylase inhibition to promote IgA class switching and anti-inflammatory Treg/IL-10 responses (Siddiqui and Cresci, 2021), aligning with the higher IgG and IgA levels observed in this study. Phytogenic actives (e.g., curcumin, capsaicin, cinnamaldehyde, and piperine) down-modulate NF-κB and activate antioxidant defenses in poultry, a profile associated with increased mucosal IgA and improved gut function (Obianwuna et al., 2024; Li et al., 2022).

The results of the present study demonstrated that coccidiosis challenge with a mixture of *Eimeria* species induced significant reductions in key morphological indices across different segments of the small intestine in broiler chickens. Notably, decreases in VH, the VH/CD ratio, VSA, and GC numbers were observed in the duodenum, jejunum, and ileum, reflecting direct intestinal mucosal damage and diminished nutrient absorption capacity. These findings are consistent with previous reports indicating that *Eimeria* infection disrupts nutrient absorption by damaging the intestinal epithelium, characterized by villus atrophy and consequent declines in growth performance and feed conversion efficiency (Suárez et al., 2023; Su et al., 2024). In contrast, phytogenic compounds and protected butyric acid, particularly in combination, improved VH, VSA, and GC density, indicating accelerated mucosal regeneration and preservation of intestinal health relative to the PC group. These results corroborate the findings of Ventura et al. (2021), who highlighted the protective effects of butyric acid in enhancing intestinal morphology and promoting mucosal repair following a coccidiosis challenge. Protected butyric acid glycerides serve as a crucial energy substrate for intestinal epithelial cells and possess anti-inflammatory properties that support mucosal barrier integrity (Kpodo et al., 2025). Their sustained release in the small intestine confers enhanced efficacy and stability against rapid metabolism, thereby facilitating more efficient local repair of *Eimeria*-induced mucosal damage (Zhou et al., 2017). Furthermore, the phytogenic supplement exerts anti-inflammatory and antioxidant effects that mitigate intestinal epithelial damage (Ogwuegbu and Mthiyane, 2024; Eldeeb et al., 2025). Curcumin and cinnamaldehyde specifically protect mucosal cells and promote villus elongation by inhibiting lipid peroxidation and reducing oxidative stress (Shi et al., 2020; Yang et al., 2021). These mechanisms enhance the VH/CD ratio and increase the absorptive surface area, thereby improving nutrient uptake and growth performance. In line with these outcomes, butyrate can directly strengthen epithelial barrier function by activating AMP-activated protein kinase (AMPK) and accelerating tight-junction assembly (e.g., ZO-1 and OCLN relocalization) in intestinal monolayers (Peng et al., 2009). Beyond barrier biology, butyrate also modulates mucosal immunity and can enhance IgA and IgG responses (Siddiqui and Cresci, 2021), providing a mechanistic rationale for the immunological improvements observed in this study.

*Eimeria* infection provokes intestinal inflammation, disrupting mucosal barrier function and increasing intestinal permeability to pathogens (Pham et al., 2021; Lin et al., 2022). In this study, phytogenic supplements and protected butyric acid demonstrated efficacy in preventing *Eimeria*-induced damage by enhancing intestinal barrier integrity. Protected butyric acid, due to its gradual release in the small intestine, plays a crucial role in maintaining barrier function and mitigating infection-induced inflammation (Ventura et al., 2021; Mátis et al., 2022). Studies indicate that butyrate treatment increases the activity of AMPK, which in turn accelerates the assembly of tight-junction proteins, thereby strengthening the intestinal barrier (Dong et al., 2023). Phytogenic compounds, owing to their anti-inflammatory and antioxidant properties, can attenuate the adverse effects of *Eimeria*-induced inflammation and promote more rapid mucosal repair (Paraskeuas and Mountzouris, 2019; Zhang et al., 2023). Based on the results of this study, JAM-2 gene expression was elevated in all supplemented groups compared to the PC group. JAM-2 is a key tight-junction protein that preserves intestinal barrier integrity by preventing the translocation of harmful substances (Bai et al., 2022). Consistently, commensal *Lactobacillus johnsonii* has been shown to reinforce barrier function through direct interaction with epithelial JAM-2, underscoring the biological relevance of JAM-family regulation to intestinal integrity (Bai et al., 2022). Notably, the combined treatment (PC+BAG+MPH) significantly increased CLDN1 and ZO-1 gene expression relative to the PC group, indicating complementary effects of phytogenic and butyric acid in enhancing intestinal health and immune function. These enhancements in barrier preservation and resistance to *Eimeria* substantially contributed to reduced infection severity and intestinal damage (Alqhtani et al., 2023b; Javanmiri et al., 2024). Within the phytogenic blend, curcumin can activate Nrf2/HO-1 antioxidative signaling in the intestine and mitigate permeability increases during enteric stressors in broilers (Zhang et al., 2024), aligning with the tighter junction phenotype we observed. Piperine can increase the effective exposure of co-ingested phytochemicals by inhibiting intestinal P-glycoprotein/CYP3A4, a well-described mechanism that may potentiate curcumin and cinnamaldehyde bioactivity at the epithelial interface (Jäger et al., 2014).

## Conclusions

This study demonstrates that dietary supplementation with multicomponent phytogenic supplement and butyrate glycerides effectively improves growth performance, intestinal integrity, and immune function in broiler chickens under a coccidiosis challenge. The combined treatment (PC+BAG+MPH) significantly improved villus morphology, upregulated tight-junction gene expression (JAM-2, CLDN1, ZO-1), and increased immunoglobulin levels (IgG, IgA, IgM), indicating strengthened gut barrier function and systemic immunity. Additionally, reduced oocyst shedding and intestinal lesion severity confirm the protective role of these additives against *Eimeria*-induced intestinal damage. These findings support the practical use of phytogenic and butyrate glycerides as natural, antibiotic-free strategies for promoting gut health and controlling coccidiosis in poultry production systems.

## CRediT authorship contribution statement

**Hossein Ali Ghasemi:** Writing – review & editing, Writing – original draft, Supervision, Investigation, Conceptualization. **Hamidreza Nahandast:** Writing – review & editing, Visualization, Methodology, Investigation, Data curation. **Iman Hajkhodadadi:** Writing – review & editing, Software, Resources, Methodology, Data curation. **Navid Nari:** Visualization, Validation, Software, Investigation, Data curation. **Kamran Taherpour:** Writing – review & editing, Writing – original draft, Resources, Project administration, Formal analysis.

## Disclosures

All authors approve the submission of this manuscript and declare no conflict of interest.

## Appendix Supplementary materials

Image, image 1

Image, image 2
