# Supplementary figures and images for: Individual and combined effects of butyric acid glycerides and a multicomponent phytogenic supplement on growth performance, intestinal mucosal health, and immune response of broiler chickens under coccidiosis challenge
Source: Poult Sci. 2025 Oct 31;104(12):106046. doi: 10.1016/j.psj.2025.106046 (PMC12639308; doi:10.1016/j.psj.2025.106046)

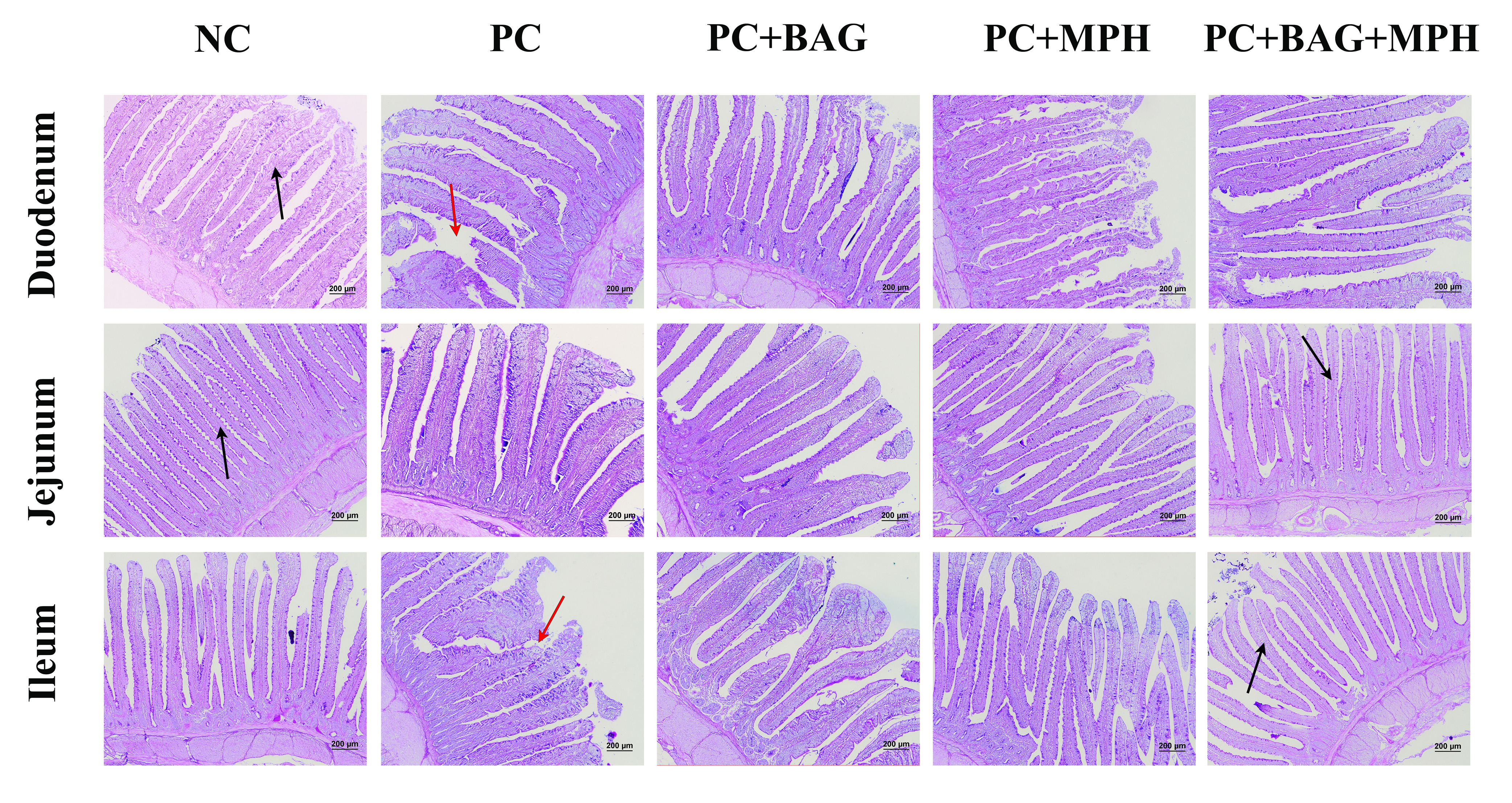

Supplement: Supplementary file 1 [file mmc1.jpg]

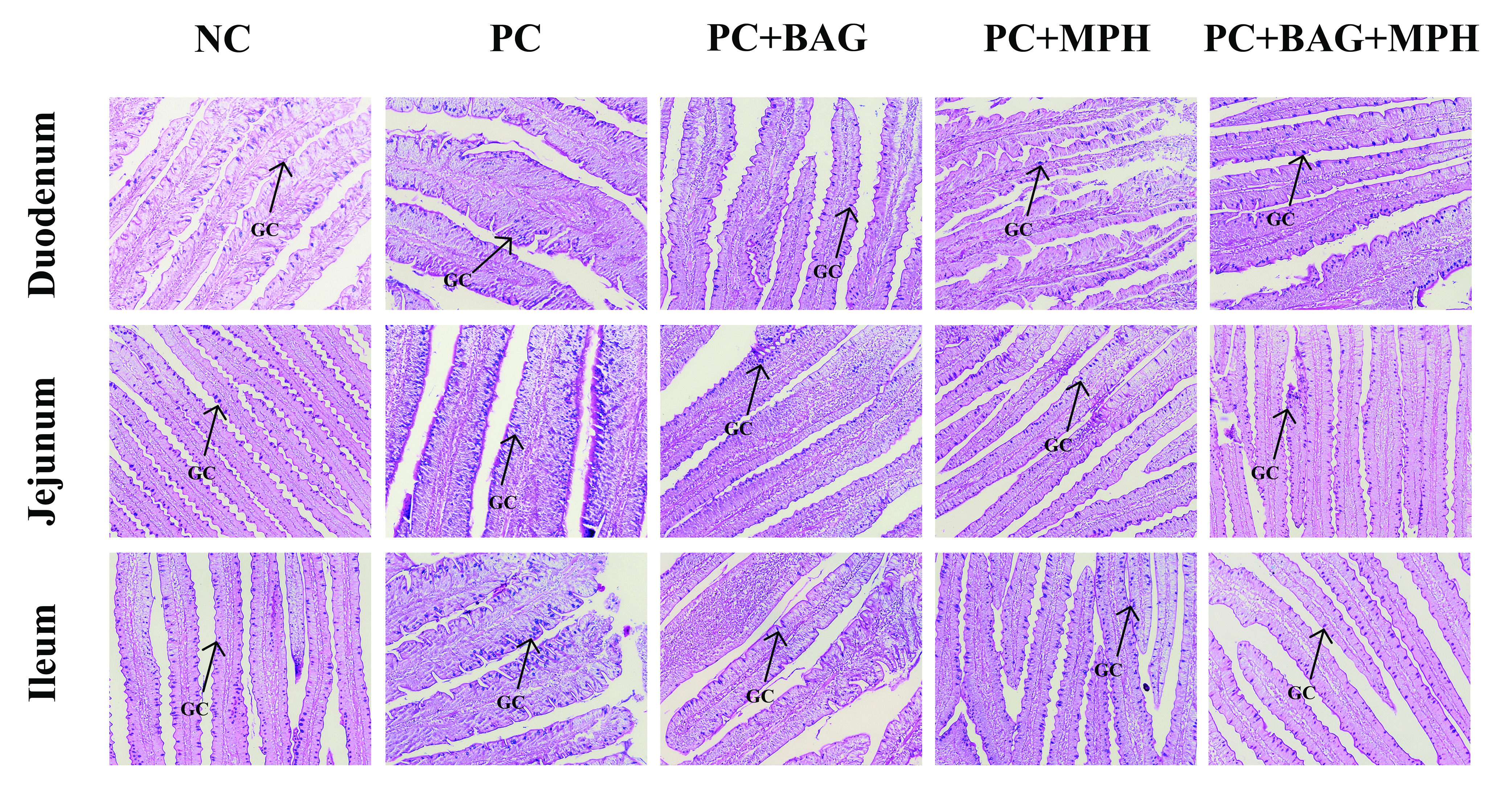

Supplement: Supplementary file 2 [file mmc2.jpg]
